# Gene Knockdowns in Adult Animals: PPMOs and Vivo-Morpholinos

**DOI:** 10.3390/molecules14031304

**Published:** 2009-03-25

**Authors:** Jon D. Moulton, Shan Jiang

**Affiliations:** Gene Tools, LLC, 1001 Summerton Way, Philomath, OR 97370 USA

**Keywords:** Antisense, Morpholino, PPMO, Vivo-Morpholino.

## Abstract

Antisense molecules do not readily cross cell membranes. This has limited the use of antisense to systems where techniques have been worked out to introduce the molecules into cells, such as embryos and cell cultures. Uncharged antisense bearing a group of guanidinium moieties on either a linear peptide or dendrimer scaffold can enter cells by endocytosis and subsequently escape from endosomes into the cytosol/nuclear compartment of cells. These technologies allow systemic administration of antisense, making gene knockdowns and splice modification feasible in adult animals; this review presents examples of such animal studies. Techniques developed with PPMOs, which are an arginine-rich cell-penetrating peptide linked to a Morpholino oligo, can also be performed using commercially available Vivo-Morpholinos, which are eight guanidinium groups on a dendrimeric scaffold linked to a Morpholino oligo. Antisense-based techniques such as blocking translation, modifying pre-mRNA splicing, inhibiting miRNA maturation and inhibiting viral replication can be conveniently applied in adult animals by injecting PPMOs or Vivo-Morpholinos.

## 1. Introduction

Morpholino oligos are steric-blocking antisense molecules which bind to RNA and get in the way of cellular processes. These oligos have no electrical charge, do not interact strongly with proteins, and do not require the activity of RNase-H, Argonaute, or other catalytic proteins for their activity. Antisense molecules however do not readily cross cell membranes without delivery techniques, which has prevented their effective use in adult animals [1]. With delivery of Morpholino oligos achieved *in vivo* by Vivo-Morpholinos and Morpholino oligos linked to arginine-rich cell penetrating peptides (PPMOs), a longstanding barrier to applying Morpholino antisense techniques in adult animals has been overcome. Vivo-Morpholinos and PPMOs enter cells from the extracellular space and gain access to the cytosol and nuclear compartments. Antisense effects can be observed after systemic delivery of Vivo-Morpholinos or PPMOs. 

### 1.1. Uses of unmodified Morpholinos

Effective techniques for delivering the oligos to the cytosol and nuclear compartments in tissue cultures have been developed, such as mechanical scraping [2], electroporation [3] or use of endosomal escape reagents [4]. Unmodified Morpholinos have been used routinely to block translation, modify splicing, inhibit miRNA activity and inhibit viral replication as well as more exotic RNA-blocking applications [5]. The Morpholino antisense structural type has been a revolutionary tool in developmental biology [6]. Success in embryos came by microinjecting Morpholino oligos into eggs or single or few celled zygotes, so that during cell division the Morpholinos were apportioned into daughter cells. This neatly avoided the problem of delivering the antisense separately into each cell of a many-celled organism [7]. In addition to their use to determine gene functions and interactions in embryos, Morpholino antisense oligos have been used for research into a broad range of diseases as well as several clinical trials (AVI BioPharma, Inc.). In most cases, carrying work from research to therapeutic applications of unmodified Morpholino oligos has been limited by difficulties with *in vivo* delivery [8]. However, delivery of relatively high doses of unmodified Morpholinos into dystrophic muscle in animal models of DMD has induced expression of functional Dystrophin in skeletal muscle [9,10] and unmodified Morpholinos are currently in clinical trial for treatment of DMD (http://clinicaltrials.gov/ct2/show/NCT00844597).

### 1.2. Morpholino chemistry and nomenclature

Morpholino oligos are manufactured from ribosides. The ribose ring is opened by oxidation, re-closed on ammonia and the product subsequently reduced to substituted morpholine. The base and the morpholine nitrogen are protected and the subunit is activated with a dimethylamino phosphoro-dichloridate. The activated subunits are added to a synthesis resin with washing, deprotection and activation steps for each activated base added. Oligos are cleaved from the resin and deprotected with ammonium hydroxide, then purified and quantitated, often followed by lyophilization and sterilization [11]. Substitutions such as peptides or the dendrimer scaffold for guanidinium may be added to the 3’ morpholine nitrogen while the oligo is still attached to the synthesis resin. Alternatively, peptides may be added in the solution phase after resin cleavage and purification steps.

The ends of a Morpholino oligo are described as 3’ and 5’, but these labels do not refer to properly numbered atoms of the Morpholino backbone. Instead the atom designations of natural nucleic acids are used by analogy to label Morpholino ends; nucleic acids have a 5’-methylene hydroxyl (often phosphorylated) at one end and a 3’-ring hydroxyl at the other end. The methylene attached to the morpholine ring is designated as the 5’ end of a Morpholino subunit and the morpholine nitrogen is designated as the 3’ end; this nomenclature is chosen in order to make designation of direction along the Morpholino oligo familiar to molecular biologists, who are accustomed to referring to the 5’ and 3’ ends of RNA and DNA.

### 1.3. Endocytosis and the barrier to entry into the cytosol/nuclear compartment

**Figure 1:**
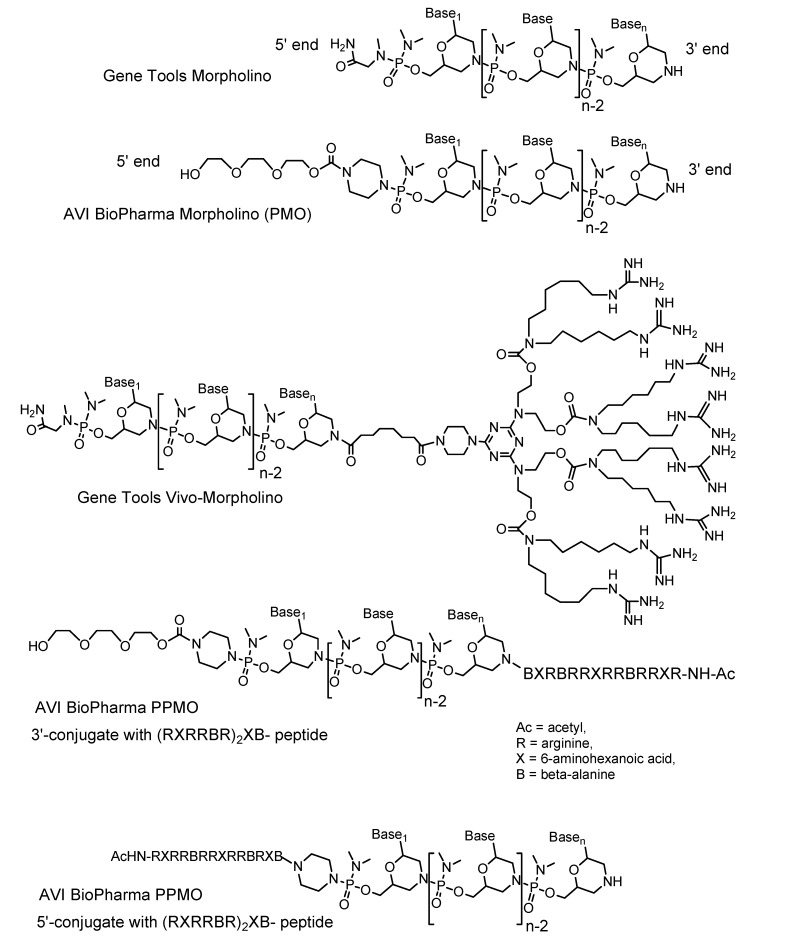
Structures of unmodified and delivery-enabled Morpholino oligos.

Unmodified Morpholino oligos are endocytosed but have little to no activity against their RNA targets. Uptake by endocytosis does not mean the antisense oligos are reaching the cytoplasm or nucleus of the cell. Fluorescently-labeled Morpholinos can be detected as dim punctuate fluorescence within endosomes, but in most cells too little antisense escapes from the endosomes to the cytosol/nuclear compartment to be biologically active [2]. Morpholinos are not degraded in endosomes [12] but remain trapped. Many biological problems are best studied in adults and so a method that allows entry of antisense into most or all cells in an adult organism is very desirable. Ideally this systemic delivery could be accomplished by routine means such as intravenous or intraperitoneal injections. A technology that allows enough of the endocytosed oligos to escape from endosomes to have biological activity would enable *in vivo* delivery of oligos administered by systemic injection. Such technologies have now been developed: PPMOs and Vivo-Morpholinos. A PPMO may have an arginine-rich cell-penetrating peptide linked to either the 3’ end or the 5’ end of the Morpholino oligo [13]. The arginines have guanidinium moieties as part of their side chains, and the presence of these guanidiniums have been shown to increase cellular uptake of conjugated materials [14]. Studies of the mechanism of PPMO entry have shown that the cell-penetrating peptides offer two advantages over unmodified Morpholinos: enhanced uptake into endosomes and, critically, enhanced ensodomal escape [15]. A Vivo-Morpholino has an octaguanidinium dendrimer constructed on the 3’ end of a Morpholino oligo [16] (Figure 1).

### 1.4. PPMOs

The first effective chemically-mediated method for systemic delivery of Morpholino antisense was based on covalently linking the oligos to cell-penetrating peptides, using arginine-rich cell-penetrating peptides to deliver uncharged antisense molecules [17]. These molecules are called peptide-linked phosphorodiamidate Morpholino oligomers, or PPMO for short. Research to develop more safe and effective peptide sequences for PPMOs has been the focus of Hong Moulton’s group at AVI BioPharma Inc. [18,19], where preclinical work on PPMOs for Duchenne muscular dystrophy is currently ongoing. Studies on PPMOs have shown that uptake of the oligos is an energy-dependant and temperature-dependant process that can be prevented using endocytosis inhibitors; these characteristics indicate that uptake of the PPMOs is by endocytosis [20]. A fraction of endocytosed PPMOs escape from the endosome, entering the cytosol and nuclear compartment where they can block mRNA translation and modify pre-mRNA splicing [21]. Intravenous injection [22], intraperitoneal injection or intranasal administration [23] of PPMOs to mice have inhibited viral replication. Injections of PPMOs into transgenic mice carrying engineered splice-reporter genes have triggered expression of green fluorescent protein throughout the tissues [24].

Positively-charged arginine amino acid residues are not electrostatically attracted to uncharged antisense such as Morpholino oligos or peptide nucleic acid (PNA) oligos, so the positively charged arginines are free to interact with membranes [25]. At the end of an arginine amino acid’s side chain is a guanidinium group, which carries the amino acid’s positive charge. This charged guanidinium group is not only attracted to a phosphate because of the phosphate’s negative charge, it can also form two hydrogen bonds with the phosphate’s oxygens. This makes the attraction of a guanidinium and a phosphate an unusually strong non-covalent interaction [26]. It may be this strong interaction that distorts the endosomal membrane and renders it permeable.

### 1.5. Vivo-Morpholinos

Vivo-Morpholinos are Morpholino antisense oligos covalently linked to a molecular scaffold that carries a guanidinium group at each of its eight tips. These custom-sequence antisense molecules enable Morpholino applications in adult animals. Vivo-Morpholinos have been shown effective in mice [27,28] and are being tested in rats, adult zebrafish and various organ explants.

To make a Vivo-Morpholino, the scaffold that will hold the guanidinium groups is added to the morpholine nitrogen at the 3’-end of a Morpholino oligo while the oligo is still bound at its 5’ end to the synthesis resin. Subsequent treatment with ammonia cleaves the Vivo-Morpholino from the synthesis resin, de-protects the Morpholino’s bases and de-protects the eight tips of the 3’-terminal scaffold. Next, treatment with *O*-methyl isourea converts the eight terminal amino groups to guanidinium groups [16].

## 2. Targets and outcomes of Morpholino experiments

Morpholino oligos bind to complementary RNA. If a molecular process normally occurs at the site where the Morpholino is bound to RNA, the Morpholino might be suitably positioned to get in the way of that process and prevent it from occurring. This mechanism is called steric blocking [29]. The effect of a Morpholino on a cell depends on where the Morpholino binds to the RNA, which determines whether it can block a process and which process it blocks. There are three common applications for Morpholino oligos: blocking translation of mRNA, modifying splicing of pre-mRNA or inhibiting miRNA activity [5]. In addition, work has been done on viral targets such as internal ribosomal entry sites [3] and cyclization sequences [30]; much of the reported work with PPMOs has involved viral targets [31]. Exotic targets have also been explored, such as binding to slippery sites to trigger ribosomal frameshifting [32] or binding to ribozymes to prevent catalytic cleavage of RNA [33].

### 2.1. Translation blocking

Translation blocking is the simplest method for knocking down gene expression with a Morpholino. When making a protein by cap-dependant translation, the small subunit of a ribosome binds to the 5’ cap of an mRNA and, along with some other proteins, forms an initiation complex. The initiation complex moves along the 5’ untranslated region (UTR) of the mRNA until a start codon is reached. At the start codon, the large subunit of the ribosome docks to small ribosomal subunit and then the process begins of linking together amino acids to form the new protein. Morpholinos that block the journey of the ribosomal initiation complex from the 5’-cap to the start codon, halting translation of the mRNA before the linking of amino acids can start. These Morpholinos can bind to RNA targets anywhere in the 5’-UTR through the start of the coding region, so long as the oligo binds onto the start codon or upstream (to the 5’ direction) of the start codon [34].

The activity of a translation blocking oligo is assayed by an immunochemical method, typically Western blotting. Halting translation of a protein will not immediately cause a detectable change in the protein’s signal on a Western blot. Some preexisting protein must be degraded over time before the protein signal decreases. The time needed between knockdown and assay varies with the protein, as different proteins have different stabilities in cells.

### 2.2. Splice modification

After a pre-mRNA is transcribed from a DNA template, it goes through processing in the nucleus to form a mature mRNA. This processing typically includes adding a 5’ cap, splicing out introns and ligating together exons, and adding a 3’ poly-A tail. Splice modification involves binding Morpholinos to targets on a pre-mRNA (an unspliced mRNA) to redirect the spliceosome to a new pattern of splicing and produce a mature mRNA sequence that differs from the sequence produced in the absence of the Morpholino. Usually Morpholinos are bound to mostly-intronic targets at the boundaries between exons and introns. These targets prevent the binding of molecules that direct the spliceosome to splice sites. Typical results of splice modification include removal of a targeted exon or inclusion of the first or last intron in the mature mRNA [35].

Another class of targets consists of the binding sites on RNA where splice-regulation proteins normally bind, such as exonic splice enhancers or intronic splice suppressors. These proteins are important in directing alternative splicing, a natural process by which cells make alternative forms of mRNA from a single gene. For some targets, preventing a splice-regulatory protein from binding is an efficient method by which Morpholino can change the pattern of RNA splicing and therefore change the sequence of a mature mRNA [10,36].

Splice modification might produce an mRNA that is expressed as an altered protein and that protein might retain some activity and might still bind antibodies that would also bind to the unaltered protein. Because of this, immunochemical techniques used to detect knockdowns by translation blocking Morpholinos often do not work when used for detecting activity of splice-modifying Morpholinos. The activity of splice-modifying oligos is typically assayed by RT-PCR from primers that bind on either side of the altered sequence. If the altered splice leaves downstream sequence in-frame, the RT-PCR product will appear at a different location on an electrophoretic gel. Activation of a cryptic splice site can result in an RT-PCR fragment of unexpected size [37]. If the altered splice shifts the downstream reading frame, appearance of a premature termination codon often results in nonsense-mediated decay of the modified RNA, so the wild-spliced RT-PCR product band might dim on the electrophoretic gel without the corresponding splice-modified RT-PCR product appearing as a new band in a different position.

### 2.3. Inhibiting microRNA

A mature micro-RNA (miRNA) is a short strand of RNA bound to a protein complex which changes the expression of other genes by mechanisms that include cleaving or inhibiting translation of the target RNAs. We will discuss miRNAs of animals; there are some differences in plants. The target RNA must have sites at least partially complementary to the miRNA. Usually (but not always [38]) the target sites for miRNAs are in the 3’-UTR of mRNAs. miRNA is formed when a short stem-loop (hairpin) with the appropriate geometry forms in a newly-transcribed RNA. In the nucleus, the hairpin is recognized and cleaved by the Drosha nuclease, which cleaves both strands of the stem and leaves a few single-stranded overhanging bases. The resulting stem-loop is exported to the cytosol where it is recognized and cleaved by the Dicer nuclease, which removes the loop and also leaves a few single-stranded overhanging bases. The resulting short double-stranded RNA is loaded onto a protein complex called the RNA-induced silencing complex (RISC), where a protein in the Argonaute family binds to one strand, called the guide strand, and cleaves the other strand. The cleaved strand dissociates and diffuses away, leaving an active miRNA-RISC complex [39].

Morpholinos can bind to the miRNA guide strand on RISC, preventing the guide strand from recognizing its targets on mRNAs [40]. Morpholinos can bind to the immature RNA hairpin, preventing Drosha from releasing the stem-loop from the rest of the transcript. Morpholinos can bind to the stem-loop, preventing Dicer from cleaving the loop from the stem [41]. A typical strategy is to design a 25-base Morpholino to bind the entire guide strand sequence (around 21 bases) and slightly into the loop sequence, allowing both inhibition of mature miRNA activity on RISC and inhibition of miRNA maturation by blocking Dicer. Another option is protecting a mRNA target. By binding to its complementary miRNA, a Morpholino blocks RISC from accessing the mRNA, thus relieving translation of the mRNA from inhibition by the miRNA and protecting the mRNA from cleavage by miRNA-directed Argonaute activity [42]. An advantage of target protection is that only the expression of the target gene is altered, while inhibiting maturation and activity of a miRNA can alter the expression of many mRNAs.

## 3. PPMOs

PPMOs are covalent conjugates of Morpholino oligos with cell-penetrating peptides. The peptide may be attached at the 3’ or 5’ end of the Morpholino oligo. Successful cell-penetrating peptides have contained arginine residues. The peptides may be composed of the alpha amino acids common in natural proteins or may contain other amino acids such as β-alanine or 6-aminohexanoic acid. Some cell-penetrating peptide sequences discussed in this section include (RXR)_4_B-, (RXR)_4_XB-, (RXRRBR)_2_XB- and (RX)_8_B-, with the dash at the peptide carboxy terminus representing the link to the Morpholino oligo. For these structures, R = arginine (L-arginine unless otherwise noted ), B = β-alanine, and X = 6-aminohexanoic acid. Chiral amino acids may be in D or L forms, with the D forms chosen to resist proteolytic degradation, as in the two terminal arginines of the peptide (DR)_2_R_2_QR_2_K_2_RF_2_C-. A procedure for solution-phase conjugation of Morpholino antisense oligos to arginine-rich peptides has been published [21].

The literature of PPMO therapeutic applications has been reviewed recently [8]. This section will focus on PPMO papers published since that review was prepared, especially seeking efficacy comparisons between unmodified Morpholinos and PPMOs and assessments of toxicity or immunogenicity of the PPMOs. Where many sequences of cell-penetrating peptides were reported in a paper, we will discuss the most effective of the peptides; for more detailed structure-activity comparisons, refer to the primary papers.

### 3.1. Screening cell-penetrating peptides in EGFP mice

A set of 14 cell-penetrating peptides were attached to the 5’ ends of Morpholinos targeting a mutant splice site in human β-globin (IVS2-654) and administered to EGFP-654 mice, which carry a transgene with EGFP interrupted by an aberrantly-spliced human β-globin mutant intron (IVS2-654). Administration of different peptides resulted in different biodistributions. Because the cell-penetrating peptide (RXRRBR)_2_XB- was effective at correcting IVS2-654 splicing in heart, diaphragm and quadriceps, important target muscles for Duchenne muscular dystrophy (DMD) treatment, and did not cause toxicity at 12 mg/kg daily for four days, it was selected for intensive study. This PPMO also modified splicing in smooth muscles of the gut [24].

To determine persistence of effects, 12 mg/kg daily for four days of the (RXRRBR)_2_XB- PPMO targeting IVS2-654 was administered to a group of EGFP-654 mice and mice were sacrificed and assessed periodically for 12 weeks. As shown by RT-PCR of cardiac muscle one day after the final injection, 90% of transcripts were splice-corrected. This decreased to 10% splice correction at six weeks. In diaphragm, splice correction decreased from 100% at 2-3 weeks after treatment to 10%-40% at 12 weeks. In quadriceps, splicing was still 100% corrected at 12 weeks. [24]

### 3.2. Duchenne muscular dystrophy

Duchenne muscular dystrophy (DMD) arises from some mutations of the human dystrophin gene. A potential treatment for this disease is the use of steric-blocking oligos to redirect splicing, skipping exons to remove early stop codons or to correct the reading frame disrupted by frameshift mutations. Several mouse models of DMD have been developed, the first and most popular being the *mdx* mouse, which has a premature stop codon in exon 23. Unmodified Morpholino antisense is currently in clinical trial for DMD. However, unmodified Morpholinos do not enter the heart at effective concentration and affect dystrophin splicing, even after repeated doses as high as 100 mg/kg in *mdx* mice [9]. For the three papers reviewed below, measurements of mRNA exon 23 skipping and Dystrophin concentrations after systemic delivery of PPMOs in *mdx* mice are summarized in Table 1.

#### 3.2.1. Studies with the (RXRRBR)_2_XB- cell-penetrating peptide PPMOs

A PPMO with the (RXRRBR)_2_XB- peptide conjugated to the 3’ end of a Morpholino sequence designed to cause splice-mediated excision of dystrophin exon 23 was injected iv into *mdx* mice at 12 mg/kg daily for four days. RT-PCR of RNA from heart tissue found that 70% of the dystrophin mRNA lacked exon 23 one day after the last treatment, decreasing to 50% at two weeks and 20% at seven weeks. At nine weeks after treatment, exon 23-skipped mRNA was still detected. In diaphragm and quadriceps, almost complete skipping of exon 23 persisted through nine weeks [24].

In-gel immunostaining revealed that in heart 30% of normal dystrophin levels were reached at 2-3 weeks and 15% remained at seven weeks after treatment. In diaphragm and quadriceps 40%-50% of normal dystrophin remained up to 17 weeks after treatment [24].

A PPMO with the (RXRRBR)_2_XB- peptide on a Morpholino targeting dystrophin exon 23 in the *mdx* mouse was compared with an unmodified Morpholino of the same sequence. Either the Morpholino or the PPMO were injected im at 30 mg/kg into the tibialis anterior (TA) of adult *mdx* mice. The PPMO induced strong dystrophin expression in 85% of the fibers of the TA while the Morpholino induced dystrophin expression in 14% of TA fibers [43].

**Table 1:** Comparison of systemic delivery of PPMOs for DMD.

PPMO with (RXRRBR)_2_XB- peptide on 3’ end [24], 12 mg/kg iv tail vein, daily for 4 d	
Dystrophin **mRNA** with exon 23 skipped	Dystrophin **protein** concentration
Heart:	Heart:
70%, 1d post-treat;	30% dystrophin 2-3wk post-treat;
50%, 2wk post-treat;	15% dystrophin 7wk post-treat.
20%, 7 wk post-treat.	Diaphragm & quadriceps:
Diaphragm & quadriceps:	40%-50% dystrophin, 17 weeks post-treat.
~100% thru 9 wk post-treat.	
PPMO with (RXRRBR)_2_XB- peptide on 5’ end [43], 30 mg/kg iv retro-orbital, single dose	
Dystrophin **mRNA** with exon 23 skipped	Dystrophin **protein** concentration
Heart:	Heart:
63%, 2wk post-treat.	58% dystrophin, 2wk post-treat.
Skeletal muscle:	Skeletal muscle:
80%-86%, 2wk post-treat.	91%-100% dystrophin, 2wk post-treat.
PPMO with (RXRRBR)_2_XB- peptide on 5’ end [43], 30 mg/kg iv retro-orbital, once every two weeks for three months (six times)	
Dystrophin **mRNA** with exon 23 skipped	Dystrophin **protein** concentration
Heart:	Heart:
72%, 2wk post-treat.	~100% 2wk post-treat.
Skeletal muscle:	Skeletal muscle:
85%-92%, 2wk post-treat.	~100% 2wk post-treat.
PPMO with (RXR)_4_XB- peptide [44], 25 mg/kg iv tail vein, single dose	
Dystrophin **mRNA** with exon 23 skipped	Dystrophin **protein** concentration
Heart:	Heart:
50%, 3wk post-treat.	10%-20% dystrophin, 3wk post-treat.
Skeletal muscle:	Skeletal muscle:
Near 100%, 3wk post-treat.	25%-100% dystrophin, 3wk post-treat.

Percent protein and mRNA are compared with wild-type muscle as 100%.

Single retro-orbital iv injections of the 30 mg/kg PPMO were administered to *mdx* mice and dystrophin expression was assessed two weeks later. Similar injections of the unmodified Morpholino induced dystrophin expression in 5% of skeletal muscle fibers. The PPMO induced strong dystrophin expression in 100% of skeletal muscle fibers and near-normal dystrophin levels were found by Western blot. RT-PCR of PPMO-treated mice showed 80%-86% of dystrophin transcripts in skeletal muscle were missing exon 23, demonstrating PPMO activity. Unmodified Morpholino induced no detectable dystrophin expression in cardiac muscle fibers. The PPMO induced dystrophin expression in 94% of cardiac muscle fibers and 58% of normal dystrophin levels was found by Western blot. RT-PCR revealed that 63% of dystrophin transcripts in cardiac muscle were missing exon 23 [43].

To assess longer-term treatment, *mdx* mice were iv injected retro-orbitally with PPMO at 30 mg/kg once every two weeks for three months, totaling six injections per mouse. Two weeks after the last injection, dystrophin was found in 100% of muscle fibers including smooth muscle of the small intestine. Dystrophin levels resembled those of normal mouse tissue. In cardiac muscle exon skipping was improved by this dosing regimen, with 72% of dystrophin mRNA lacking exon 23 and strong dystrophin protein expression comparable to normal heart [43].

No damage to kidney or liver was detected by histological exams after PPMO treatment. Alkaline phosphatase and creatinine were not altered by PPMO treatment. Inflammatory cells did not accumulate in muscles and no antibodies reactive with PPMO were found in serum, indicating that the PPMO was not immunogenic [43]. 

#### 3.2.2. Study with the (RXR)_4_XB- cell-penetrating peptide PPMO

Single tail vein injections of 25 mg/kg PPMO with peptide sequence (RXR)_4_XB- and a Morpholino sequence designed to cause excision of exon 23 were administered to *mdx* mice. Three weeks later, skeletal muscles were immunostained and showed near normal levels of dystrophin in most muscles analyzed. Western blot analysis found dystrophin concentrations ranging between 25% to 100% of normal in skeletal muscles. RT-PCR revealed almost total skipping of exon 23 in all skeletal muscles analyzed [44].

In heart muscle RT-PCR showed that about 50% of dystrophin transcripts lost exon 23. Immunostaining revealed dystrophin-positive fibers were widely distributed. By Western blotting, dystrophin concentrations between 10% and 20% of normal levels in heart were found [44].

PPMOs with the cell-penetrating peptides (RXR)_4_XB- and (RXRRBR)_2_XB- were injected iv and their efficacy compared. While both delivered antisense activity to skeletal and cardiac muscle, the (RXR)_4_XB- did so more effectively [44].

To detect toxicity, analysis of the histology of liver and kidney and assays of some serum components were performed on samples from *mdx* mice that had been treated with 25 mg/kg PPMO. No overt signs of tissue damage in kidney or liver were revealed in haematoxylin and eosin stained tissues and there was no change in the number of infiltrating cells compared with untreated *mdx* mice. In the PPMO treated mice, levels of aspartate aminotransferase (AST) and alanine aminotransferase (ALT) went from levels typical of *mdx* mice to levels typical of normal control mice. Serum levels of urea and creatinine did not change with PPMO treatment [44].

Weekly 6 mg/kg tail vein injections of the same PPMO for three weeks resulted in less exon skipping than the single 25 mg/kg tail vein injections, with the clearest decreases in efficacy in diaphragm, abdominal wall muscles and heart [44].

### 3.3. β-thalassemia

β-thalassemia is a common inherited genetic disease of humans. The ability of PPMOs to redirect splicing in a specific splice mutant of human β-globin was assessed in heterozygous knock-in mice, in which two adult murine β-globin genes (β-major and β-minor) were deactivated. This study used the cell-penetrating peptide (DR)_2_R_2_QR_2_K_2_RF_2_C- covalently linked to a Morpholino targeting the aberrant 5’ splice site created by the mutation IVS2-654 (C>T). Blocking this splice site restores normal splicing of β-globin transcripts bearing this IVS2-654 (C>T) mutation. Mice were treated with 25 mg/kg injections with daily injections for four days followed by three rest days, then this cycle was repeated. The experiment terminated on day 19 [45].

No significant liver or kidney toxicity were detected in PPMO-treated mice compared to saline-treated mice by standard biochemical tests (AST, ALT, BUN, CREA), nor was there significant weight loss. Inflammatory response was assessed by RT-qPCR of IFN-γ and LI-12a cytokines; there was no significant change detected in their expression comparing saline-injected with PPMO-injected mice. Treatment of mice with a boost of PPMO a month after they were initially primed with 12 to 16 doses of the PPMO did not result in an IL-12 response as assessed by immunoassay for IL-12 on sera from days 3, 7 and 28 after boosting. Remaining sera from days 3, 7 and 28 after boosting was tested by ELISA for antibodies to the cell-penetrating peptide; no such antibody was detected. Lymphocytes from mice that had been treated 10 months earlier with either saline or PPMO were cultured ex vivo, challenged with PPMO and tested for induction of IFN-γ or antibody to the cell-penetrating peptide; neither response was detected [45].

The level of β-globin restoration theoretically achievable under these experimental conditions was fairly low. First, the mice were heterozygous for the human knock-in, so the human hemoglobin could only be restored to 50% normal concentration from the human gene. The duration of the experiment was 19 days but murine erythrocytes normally reside in blood for 60 days, so only a maximum of 1/3 of the erythrocytes could be replaced by the end of the experiment. Combining this with the 50% expression of human β-globin from the heterozygotes, the fraction of hemoglobin containing human β-globin theoretically achievable by the end of the experiment drops just under 17%. The level of chimeric mouse-human hemoglobin reached about 1-5% of the total hemoglobin in treated mice by day 19 [45].

### 3.4. Antiviral applications

Typically the highest antiviral efficacies are achieved with pre-infection administration of the PPMO followed by a series of post-infection doses. Recent studies explicitly comparing effects of unmodified Morpholino oligos and PPMOs show that the PPMOs are far more effective *in vivo*. Viral applications of PPMOs have recently been reviewed [31,46] so only a few recent papers applying PPMOs to inhibit viruses will be considered here.

#### 3.4.1 Dengue study, mice and cultured cells, 2008

PPMOs in this study used the (RXR)_4_B- cell-penetrating peptide moiety. Efficacy of Morpholinos and PPMOs targeting Dengue virus were tested in Vero cells and in AG129 mice. Oligos targeting the 5’ terminal nucleotides (5’SL) or the 3’ cyclization sequence (3’CS) were tested along with scramble-sequence control oligos. PPMO with Morpholino sequences ranging from 14 to 30 bases were prepared and tested in Vero cells against the virus DENV-2 D2/IC-30PA; PPMOs with Morpholino components of 22-24 bases length most effectively decreased viral titer. When the effect of oligo length on PPMO uptake was tested by flow cytometry of cells treated with PPMOs of 18, 24 or 30 base length bearing fluorescent labels, no effect of length on uptake was shown and PPMOs of all lengths tested entered over 95% of the cells. Toxicology and pharmacokinetic studies on PPMOs were included with this study of antiviral efficacy of Morpholinos and PPMOs against Dengue virus. Dose regimens were tested for toxicity in AG219 mice before being used in separate AG219 mice to assess Dengue inhibition. Pharmacokinetic measurements and additional toxicity tests were done using BALB/c mice [47].

AG219 mice were treated by ip injection with daily doses of 380 μg of scramble-sequence Morpholino oligo (about 19.1 mg/kg) or 240 μg of scramble-sequence PPMO (about 12.1 mg/kg) for two days, followed by monitoring over 35 days. No ill effects were observed and the animals maintained their weights. The same dosing regimen was used with oligo sequences targeting Dengue virus but between the two oligo doses the mice were infected with 10^6^ pfu DENV-2 NGC. Treatment with 240 μg of a 5’SL-targeted 20-mer PPMO extended average survival by eight days and treatment with 240 μg of a 3’CS-targeted 18-mer PPMO extended average survival by five days when compared with control mice which were similarly infected but treated with PBS and averaged nine days survival. There was no survival benefit when mice were treated with 380 μg of unmodified Morpholino oligo targeting either 5’SL or 3’CS, nor after Morpholino or PPMO scramble-sequence treatment [47].

To determine toxicity of a longer dosing regimen, AG219 mice were treated with 10 mg/kg daily ip injection of 5’SL 22-mer for nine days and compared with a group receiving similar PBS injections. The animals increased in mass by an average of 1 g over the 35-day study regardless of treatment arm, with all animals maintaining very similar weights. This experiment was repeated but with 10^4^ pfu Dengue infection between the second and third oligo doses. Treatment with a combination of 5’SL 22-mer and 3’CS 22mer extended life eight days on average and treatment with 5’SL 22-mer extended life seven days on average, while other treatments did not extend life by statistically significant periods compared to the 14 day survival of PBS-treated infected controls [47].

BALB/c mice were treated with fluorescein-conjugated PPMO 5’SL 24-mer in an intraperitoneal dose of 10 mg/kg. Pharmacokinetics and biodistribution into brain, liver and spleen were determined from measurements at 0.2, 0.5, 1, 2, 3, 8, 12, and 24 hours. Peak plasma concentration was 0.48 mg/L. A distribution half-life of 2.79 h and an elimination half-life of 7.31 h were determined. The liver accumulated the most PPMO-Fl the most rapidly while the brain accumulated the least. Toxicity in Balb/c mice was addressed for a nine dose regimen with daily ip injection of 10 mg/kg fluoresceinated 5’SL 24-mer PPMO. Animals appeared and behaved normally through the nine-day dosing and five- day washout period. Significant increase in serum cholesterol and decreases in haematocrit, haemoglobin, packed cell volume and red blood cell count were observed during treatment but returned to levels similar to control mice during the washout period. Total bilirubin did not increase during treatment, so treatment likely inhibited red blood cell production instead of increasing red blood cell lysis. The concentrations of fluoresceinated PPMO in liver and spleen did not change significantly over the washout period, demonstrating good retention in tissues after uptake [47].

#### 3.4.2. Ebola study, mice and cultured cells, 2009

This study used oligos complementary to the ebola Zaire virus VP24 transcript, comparing unmodified Morpholinos and PPMOs with a different class of modified Morpholinos bearing a cationic moiety on some of their phosphorodiamidate linkages (PMO+). PPMOs in this study used several cell-penetrating peptide moieties, including (RXR)_4_XB-, (RX)_8_B- and (RB)_8_B-. The PPMOs were shown to have higher efficacies than PMO+ or unmodified Morpholinos against ebola virus when tested in a cell free translation system, Vero E6 cell cultures and C57BL/6 mice. Some unmodified Morpholino sequences blocked translation in cell-free translation systems but these generally had no or limited efficacy in cell cultures or in mice [48].

Vero E6 cells were pre-treated with oligo for two hours, then infected with a GFP-expressing Zaire ebola virus. After 48 hours the cells were fixed and observed for fluorescence. Uninfected cells had very little autofluorescence. Infected untreated cells and infected cells pre-treated with bare Morpholinos were clearly fluorescent. Infected cells pre-treated with PPMO had reduced fluorescence, and PPMO with peptide conjugated at the oligo’s 5’ end reduced fluorescence more effectively than PPMO with the peptide attached to the 3’ end of the Morpholino moiety. Three different cell-penetrating peptides were tried, linked at the 5’ end and the 3’ end of the Morpholino; at 10 μM all of the PPMOs with 5’-linked cell-penetrating peptides inhibited viral replication more than 35% when compared to infected cells treated with unmodified Morpholino [48].

Mice were treated twice with PPMOs having (RX)_8_B- peptide conjugated to Morpholinos targeting the 5’-terminal end of the VP24 transcript. Mice were injected at 24 hours prior and at four hours prior to infection with ebola virus. Lethal challenges of 1000 pfu ebola virus were delivered ip and the mice were tracked for 15 days. The PPMOs having (RX)_8_B- peptide conjugated to the 5’ end of the Morpholino were an effective treatment as doses of 50 μg or 5 μg kept all treated mice alive and doses of 1 μg saved 60% of mice tested. In a similar experiment with pretreatment at 24 hours prior and four hours prior to infection with 1000 pfu ebola virus ip, PPMOs constructed with fewer RX repeats were not as effective at protecting mice from ebola challenges and unmodified Morpholinos did not prevent death of the mice when used in this experiment at 50 μg per injection. Similar experiments using PPMOs having (RX)_8_B- peptide conjugated to Morpholinos targeting the start codon of the VP24 transcript gave 100% protection at 50 μg per dose and 60% protection at 5 μg per dose when the oligos were conjugated at their 5’ end and gave 95% protection at 50 μg per dose and 95% protection at 5 μg per dose when the oligos were conjugated at their 3’ end. In this experiment, unmodified Morpholinos gave 15% protection at 50 μg per dose [48].

#### 3.4.3. Influenza study, mice, 2008

Morpholinos conjugated with the (RXR)_4_XB- cell-penetrating peptide were administered intranasally to Balb/C mice and inhibited viral replication. One oligo targeted the start site of viral polymerase subunit PB1 mRNA (PB1-AUG), while another targeted the 3’ end of the NP virion RNA (NP-V3’); these were prepared as unmodified Morpholinos and as PPMOs. Intranasal delivery to the lung tissue was shown effective using a scrambled-sequence PPMO conjugated with fluorescein, by which delivery into the lung was shown to be best in the upper lung and closest to the major bronchioles and decreasing through the middle and into the lower lung. Toxicity testing was performed for treatment of up to 7.5 mg/kg administered intranasally once and again 24 hours later. Compared to saline-treated mice, PPMO-treated mice showed no significant weight loss, assessed by daily weighing over the 72 hours following the first dosing, and no observable illness. No significant differences were noted from histopathological examination of lung tissue compared to saline-treated mice. Leukocyte populations in spleen and lungs were followed by flow cytometric analysis; no significant changes in spleen populations were found and lung populations only differed significantly at the highest dose per administration (7.5 mg/kg, with no significant differences at doses of 3.75 mg/kg or less). At 7.5 mg/kg dosing, macrophages increased 2.21 fold and granulocytes increased 3.38 fold. The authors suggest that the arginine-rich cell-penetrating peptides may be causing leukocyte infiltration by mimicking a known effect of poly-arginine, which triggers a response similar to major basic protein, a component of the granules of eosinophils [49].

To test antiviral activity, mice were dosed intranasally with a 50:50 mixture of the two PPMOs, PB1-AUG and MP-V3’, at 3.74 mg/kg in each dose administered at four hours before and 20 hours after infection with influenza virus A/Eq/Miami/1/63 (H3N8). These mice were compared with mice dosed similarly with unmodified Morpholinos, with Morpholino and PPMO scramble sequence controls, or with saline. Lung tissue was taken at 72 hours after infection for plaque assays and the mixed PB1-AUG and MP-V3’ PPMO was shown to reduce viral growth 1.5 log_10_ compared to scramble PPMO. The mixed PB1-AUG and MP-V3’ unmodified Morpholinos caused slight but not significant decrease in viral titer compared to the scramble Morpholino. Tests comparing pre-infection treatment versus post-infection treatment showed clear benefit to pre-treatment, though smaller but still significant decreases in viral titer occurred with treatment delayed to 1 hr or 2 hr post-infection. [49]

### 3.5. Restenosis

Global Therapeutics, the cardiology unit of Cook Medical (http://www.cookmedical.com), is developing PPMO-coated stents for balloon angioplasty using PPMOs from AVI BioPharma Inc. The Morpholino moiety targets c-myc to inhibit proliferation of the vascular endothelial cells, which might otherwise cause restenosis.

## 4. Applications of Vivo-Morpholinos in animal studies

Vivo-Morpholinos are Morpholino oligos coupled with eight guanidinium head groups on dendrimer scaffolds that enable delivery into cells [16]. We and others have demonstrated that like PPMOs, Vivo-Morpholinos extend proven Morpholino antisense technology to research in adult animals. Potent antisense effects were observed in a wide range of tissues in animals injected with Vivo-Morpholinos at dosages that did not produce detectable toxicity [27,28]. Vivo-Morpholinos have been used successfully in various adult animals including healthy mice [27], a mouse model of Duchenne muscular dystrophy [27,28] and a dog model of Duchenne muscular dystrophy (Toshifumi Yokota, personal communication). Vivo-Morpholinos provide powerful tools for gene regulation studies in animals with an efficient synthesis designed for high-throughput production protocols [16].

### 4.1. Dramatically improved delivery of Vivo-Morpholinos versus unmodified Morpholinos in adult animals

Morpholino oligomers have been widely used to modulate gene expression in cell cultures and in embryos of zebrafish, frogs, tunicates, chicks and sea urchins [50]. Activity of unmodified Morpholino oligos in adult animals has been reported primarily in leaky tissues such as the damaged muscles in animal models of DMD [9,10,51]. However, the delivery efficiency is highly variable within tissues and inefficient in some tissues including the heart and diaphragm muscles [9]. Vivo-Morpholinos improved delivery from blood into the cytosol and nuclei of dystrophic mice more than 50 fold compared with unmodified Morpholinos, which only entered muscle at all due to the leakiness characteristic of DMD muscle [28]. In addition, the antisense effects of Vivo-Morpholinos were achieved evenly and efficiently in body-wide tissues including heart, diaphragm and smooth muscle [28]. In healthy adult animals, iv treatment with unmodified Morpholino oligomers does not cause antisense activity in cells [27]. Recent studies have shown that Vivo-Morpholinos achieve delivery in a wide variety of tissues in normal adult mice while no detectable delivery of unmodified Morpholino was achieved at the same dosage [27]. In summary, Vivo-Morpholinos exhibit significantly higher delivery efficiency in adult animals than do unmodified Morpholinos. 

### 4.2. Animal systems tested

The efficacy of antisense effect mediated by Vivo-Morpholinos has been tested in healthy adult mice and a DMD disease mouse model with leaky muscles. Vivo-Morpholinos were tested in adult mice with intact cell membranes bearing a transgene with a mutation preventing the production of enhanced green fluorescent protein (EGFP). This error could be corrected by redirecting splicing with a Vivo-Morpholino oligo, resulting in the expression of EGFP. Recent studies demonstrated that Vivo-Morpholinos efficiently corrected the error in various tissues of the EGFP transgenic mice and showed no detectable toxicity [27]. In the *mdx* mouse model of DMD, Vivo-Morpholinos have shown potent antisense effects in body-wide muscles including the hard-to-deliver heart and diaphragm muscles [27, 28]. Vivo-Morpholinos have been tested by local injection in a DMD disease dog model in which they exhibit similar potency as when locally injected into *mdx* mice (Toshifumi Yokota, personal communication).

### 4.3. Delivery method and efficacy in different tissues

Different routes of injection result in various biodistributions of Vivo-Morpholinos to tissues. Both local and systemic delivery methods have been tested in adult mice (Table 2) [27,28]. 

**Table 2:** Tissues that were tested for antisense effect in mice injected with Vivo-Morpholinos by different routes.

Injection Method	Effective tissues	Ineffective tissues	Reference
Subcutaneous (sc)	Skin	Skeletal muscles, heart	Jiang S, unpublished data
Intramuscular (im)	Muscles near injected site	Heart, diaphragm	[28]
Intraperitoneal (ip)	Skeletal muscles: diaphragm, abdominal, limb	Heart	[28]
Intravenous (iv)	Liver, small intestine, colon, skeletal muscles, spleen, lung, heart, skin, stomach, kidney.	Brain	[27, 28]

Subcutaneous (sc) injection is mainly effective for targeting skin and fails to deliver to the muscles and heart (Jiang S *et al*. manuscript in preparation). Intramuscular (im) injection efficiently delivers Vivo-Morpholinos to the muscles near the injected sites, but not in most other tissues including the heart, brain and diaphragm. Intraperitoneal (ip) injection is the most efficient route to deliver Vivo-Morpholinos to the diaphragm and the abdominal muscles [28]. By intravenous (iv) injection, systemic delivery was achieved in most tissues including liver, small intestine, colon, skeletal muscles, spleen, lung, heart, skin, stomach and kidney but not brain [27,28].

### 4.4. Vivo-Morpholinos in DMD studies and clinical relevance

The antisense efficacy of Vivo-Morpholinos was demonstrated in several Duchenne muscular dystrophy (DMD) disease animal models [27,28]. Recent data suggest that the efficacies of Vivo-Morpholinos and PPMOs are similar after local injections in dystrophic dogs (Toshifumi Yokota, personal communication). A Vivo-Morpholino targeting a splice site on the dystrophin mRNA restored Dystrophin protein production in *mdx* mice [27]. The efficiency of antisense effect was dosage dependent. Vivo-Morpholinos exhibit significantly higher impact on both splice-modified mRNA and protein levels at a dose of 25 mg/kg than at 12.5 mg/kg [27]. A single intravenous injection of 6 mg/kg Vivo-Morpholino spliced out the defect of the dystrophin gene in more than 50% of the skeletal muscle fibers of *mdx* mice [28]. Repeated injection of 6 mg/kg Vivo-Morpholinos into *mdx* mice biweekly over 10 weeks restored Dystrophin protein to near normal level in all skeletal muscles tested including biceps, diaphragm, intercostals, tibialis anterior, quadriceps, gastrocnemius, and abdominal muscles. Significantly, these injections of Vivo-Morpholinos achieved cardiac delivery and enabled Dystrophin protein production in the heart [28]. Assays of blood markers for liver toxicity (AST,ALT) showed no change of liver function [27] and no significant impact on the body weight or the histology of the liver and kidney [28]. Importantly for potential therapeutic applications, multiple injections of Vivo-Morpholinos did not stimulate an adaptive immune response in mice as determined by ELISA against Vivo-Morpholinos using mouse serum [28]. The potent antisense effect in animals and lack of obvious toxicity at the effective dosage make Vivo-Morpholinos good research reagents for modifying gene expression. The therapeutic index and long term toxicity must be assessed because periodic administration of Vivo-Morpholinos is often required to maintain the antisense effect.

## 5. Conclusions

Until recently, effective use of Morpholino antisense has been limited to applications where delivery techniques have been devised. There have been many attempted but few successful *in vivo* applications of unmodified Morpholinos in adult animals. Development of Morpholino oligos conjugated to cell-penetrating peptides (PPMO) has made adult animal experiments feasible for Morpholinos. Clinical development of PPMOs on stents to inhibit restenosis after balloon angioplasty is expected to begin imminently and PPMOs are in late preclinical development for Duchenne muscular dystrophy. Unlike PPMOs, Vivo-Morpholinos are commercially available as research reagents. Vivo-Morpholinos have been successfully tested in adult animals, providing investigators with convenient access to research-grade delivery-enabled Morpholino antisense.
